# Effect of Herbicides on the Management of the Invasive Weed *Solanum rostratum* Dunal (Solanaceae)

**DOI:** 10.3390/plants10020284

**Published:** 2021-02-02

**Authors:** Jackline Abu-Nassar, Maor Matzrafi

**Affiliations:** Department of Plant Pathology and Weed Research, Newe Ya’ar Research Center, Agricultural Research Organization (ARO), Ramat Yishay 30095, Israel; jackline@volcani.agri.gov.il

**Keywords:** alternative weed management, buffalobur, crop and herbicide rotation, herbicide efficacy, surfactant

## Abstract

*Solanum rostratum* Dunal is an invasive weed species that invaded Israel in the 1950s. The weed appears in several germination flashes, from early spring until late summer. Recently, an increase in its distribution range was observed, alongside the identification of new populations in the northern part of Israel. This study aimed to investigate the efficacy of herbicide application for the control of *S. rostratum* using two field populations originated from the Golan Heights and the Jezreel Valley. While minor differences in herbicide efficacy were recorded between populations, plant growth stage had a significant effect on herbicide response. Carfentrazone-ethyl was found to be highly effective in controlling plants at both early and late growth stages. Metribuzin, oxadiazon, oxyfluorfen and tembutrione showed reduced efficacy when applied at later growth stage (8–9 cm height), as compared to the application at an early growth stage (4–5 cm height). Tank mixes of oxadiazon and oxyfluorfen with different concentrations of surfactant improved later growth stage plant control. Taken together, our study highlights several herbicides that can improve weed control and may be used as chemical solutions alongside diversified crop rotation options. Thus, they may aid in preventing the spread and further buildup of *S. rostratum* field populations.

## 1. Introduction

The family of *Solanaceae* counts many species widespread worldwide, either wild or cultivated [1]. These species are of international importance and numerous studies related to their biology and ecology as crops and weeds have been conducted. Several of these species, such as the subgenus of prickly nightshades (*Solanum* spp.: subgenus *Leptostemonum*) are considered as significant invasive species in many parts of the world [2,3]. Originated from of the southeastern US, these species include some of the most noxious weeds worldwide [3]. Two prickly nightshades, *S. elaeagnifolium* Cav. and *S. rostratum* Dunal, invaded Israel in the 1950s [4]. These troublesome weeds infest pastures, crops, roadsides and natural habitats. Their distribution range is quite different as *S. elaeagnifolium* has become widely spread, while *S. rostratum* remains of limited dispersal. However, in the last 10 years the distribution of *S. rostratum* increased, especially in agricultural habitats.

*S. rostratum* (common name buffalobur) is a native species of the Mexican highlands [5] which has invaded several countries worldwide, including Canada, China, Russia and Australia [6,7]. *S. rostratum* is a diploid, summer-annual, self-compatible weed species with prickles on both leaves and stems. It is a noxious weed, as it grows aggressively following habitat disturbance [6], and livestock abstains from grazing on vegetation where it grows as thorns cover the entire plant. In minor crops, such as watermelon (*Citrullus lanatus* Thunb.), onion (*Allium cepa* L.), chickpea (*Cicer arietinum* L.) and tomato (*Solanum lycopersicum* L.), for which chemical control options are poor and hand weeding is common practice, *S. rostratum* thorns may increase the difficulty of this task.

**Figure 1:**
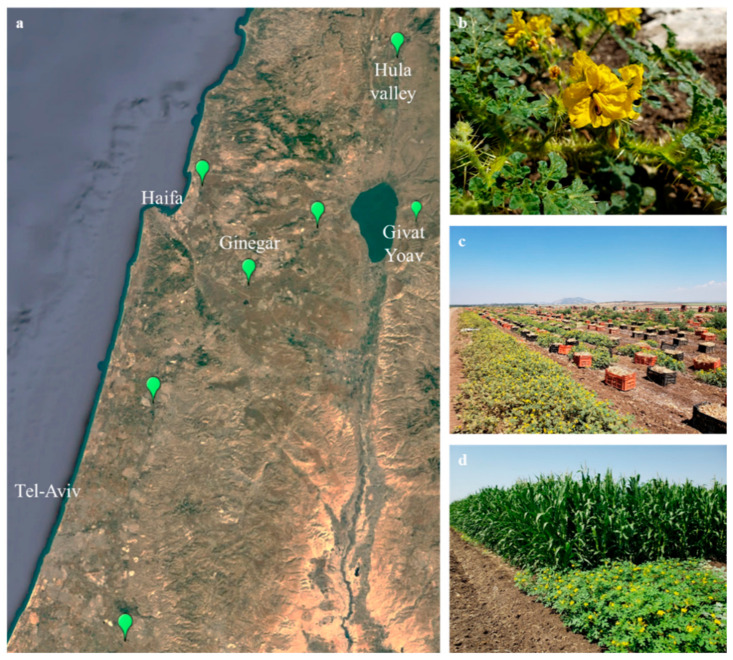
Distribution and characterization of *Solanum rostratum* at different habitats across Israel. (**a**) Geographical distribution of major *Solanum rostratum* populations. (**b**) View of mature *Solanum rostratum*. Representative photos of *S. rostratum* plants in onion (**c**) and corn (**d**) fields.

Although *S. rostratum* is highly abundant in China, it is usually found in open, disturbed habitats, such as roadsides, fallow fields and field margins [6]. In the US, this weed was reported as an agricultural pest in Oklahoma [8], Nebraska and Wyoming [9]. Field experiments conducted in cotton (*Gossypium hirsutum* L.) fields in Oklahoma showed that crop plant heights were decreased at greater densities of *S. rostratum* [8]. In the same study, yield reduction was also observed, with a damage of 480 kg ha^−1^ at a weed density of 64 plants/10 m row. In Israel, *S. rostratum* was first documented at the Jezreel Valley in 1953 [4]. Since then, several field populations were located in the Jordan Valley, the Golan Heights, the Hulla Valley and at the Mediterranean Sea coastline (Figure 1). *S. rostratum* may appear in several germination flashes, starting from spring and continuing throughout the summer; thus, both young and mature plants may coexist at the same field. Different sensitivity levels between young and mature *S. rostratum* plants can reduce the effectiveness of chemical weed control.

Studies relating to *S. rostratum* management approaches are scarce, warranting research into optimal weed control strategies, especially herbicide application. In two field experiments conducted at Nebraska and Wyoming, broadleaf weed control was tested as part of an overall strategy to reduce *S. rostratum* infestation in two summer cereal crops, proso (*Panicum miliaceum* L.) and foxtail millet (*Setaria italica* P. Beauv.) [9]. Treatments with carfentrazone-ethyl [protoporphyrinogen oxidase (PPO) inhibitor (group E), combined with either 2,4-D amine (auxin inhibitor; group O) or prosulfuron (acetolactate synthase inhibitor; group B), were highly effective in controlling *S. rostratum* plants. In Israel, PPO inhibitors such as oxyfluorfen and oxadiazon are registered for use in both onion and tomato, while carfentrazone-ethyl is registered for use in chickpea. Although the oxyfluorfen or oxadiazon herbicide labels do not require the addition of a surfactant, previous research suggested that the effectiveness of PPO inhibitors may be ameliorated by the addition of a nonionic surfactant to the tank mix [10,11].

Due to the recent increasing infestation of *S. rostratum* plants in several fields across Israel (Figure 1), this research aimed to evaluate the efficacy of herbicides with differing modes of action for the control of *S. rostratum* at different growth stages. In addition, it assessed the additive effect of surfactant to PPO inhibitors in *S. rostratum* control protocols.

## 2. Results

### 2.1. Herbicide Response of Ginegar (GO) Plants Treated at Different Growth Stages

**Figure 2:**
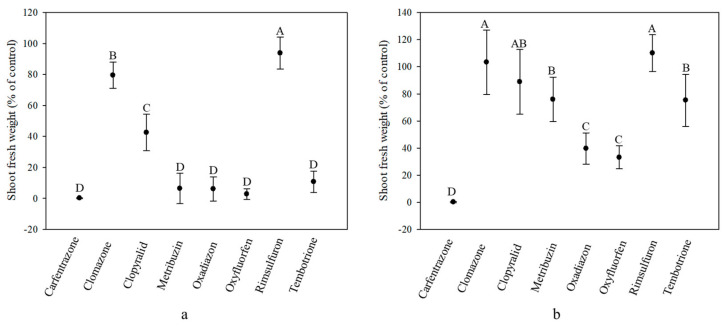
Shoot fresh weight (% of control) of *Solanum rostratum* plants from the Ginegar population treated with the recommended field rate of various herbicides at the 4–5 cm (**a**) and 8–9 cm (**b**) growth stages. Shoot fresh weight was recorded 21 days after herbicide application. Different uppercase letters indicate statistically significant differences among treatments, as determined by a Tukey–Kramer HSD test (α = 0.05).

The comparison of fresh final shoot weight indicated that plant response was significantly affected by both the specific herbicides and growth stage of the weed at the time of application. For plants that were sprayed at the 4–5 cm growth stage, both clomazone and rimsulfuron showed low efficacy in reducing plant final weight, with mean shoot fresh weights measuring 79.5% and 93.8%, respectively, of the untreated control (Figure 2a; Table 1). Clopyralid showed a more significant effect, as presented by a shoot fresh weight of 42.6% that of untreated plants. For plants treated at the 8–9 cm growth stage, low efficacy was recorded for almost all tested herbicides, with the exception of carfentrazone-ethyl, which reduced mean shoot fresh weight down to 0.2% of that of the control plants (Figure 2b; Table 1). Oxadiazon and oxyfluorfen applied at this same growth stage were significantly less effective in controlling GO *S. rostratum* plants, reducing final shoot fresh weight down to 39.7% and 33.1%, respectively, of untreated control plants. Metribuzin and tembutrione showed the same response as oxadiazon and oxyfluorfen and, while achieving high control at the 4–5 cm growth stage, at the 8–9 cm growth stage, the two herbicides provided poor weed control.

### 2.2. Herbicide Response of Givat Yoav (GY) Plants Treated at Different Growth Stages

**Figure 3:**
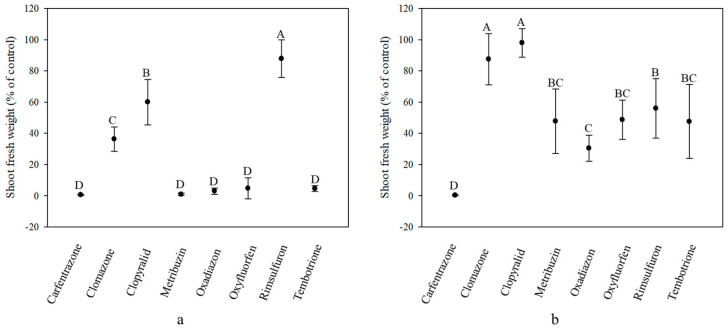
Shoot fresh weight (% of control) of *Solanum rostratum* plants from the Givat Yoav population treated with the recommended field rate of various herbicides at the 4–5 cm (**a**) and 8–9 cm (**b**) growth stages. Shoot fresh weight was recorded 21 days after herbicide application. Different uppercase letters indicate statistically significant differences among treatments, as determined by a Tukey–Kramer HSD test (α = 0.05).

The same trend of herbicide response was recorded for plants of the GY population (Figure 3a,b). As in the case of GO plants, clomazone, clopyralid and rimsulfuron, showed low efficacy on GY plants treated at the 4–5 cm growth stage. However, in contrast to the GO responses, clomazone was more effective than clopyralid, as exhibited by the mean shoot fresh weight values of 36.5% vs. 60.1%, respectively, as compared to untreated controls (Table 1). Plants treated at the 8–9 cm growth stage showed low response to herbicides, with mean shoot fresh weights not dropping below 40% for most treatments (Figure 3b; Table 1). The only herbicides that induced shoot fresh weight reductions below 40% were the two PPO inhibitors, oxadiazon and carfentrazone ethyl (30.5% and 0.3%, respectively).

### 2.3. Herbicide Effect on Plants Treated at Different Growth Stage

A negative correlation between plant growth stage and herbicide efficacy was recorded in both GY and GO populations for all herbicides, except for carfentrazone-ethyl (Table 1), with lower control rates observed when plants were sprayed at later growth stage (8–9 cm). However, the GY population response to rimsulfuron showed an opposite trend when plants were treated at the 8–9 cm growth stage; plants exhibited lower shoot fresh weight as compared to plants treated at the 4–5 cm growth stage.

### 2.4. Variation in Herbicide Response among Populations

For most herbicides, no significant differences in plant response were recorded at the 4–5 cm growth stage (Table 1). Clomazone was more effective in controlling plants from the GY population, while clopyralid was more effective in controlling plants of the GO population. However, while differences between populations, overall, both herbicides showed poor performances in controlling GO and GY plants at the 4–5 cm growth stage (clomazone 79.5% vs. 36.4% and clopyralid 42.5% vs. 60.1%, respectively). For the 8–9 cm growth stage, differences among populations were more significant, with metribuzin, oxyfluorfen rimsulfuron and tembutrione all showing differences in control efficacy of GO vs. GY plants. Although differences were significant, herbicide efficacy rates were still low, as shown by the high mean shoot fresh weight (Table 1).

### 2.5. Synergism of the Surfactant with PPO Inhibitors

The addition of surfactant to the herbicide treatments resulted in an increased sensitivity of the large size (8–9 cm) *S. rostratum* plants to both oxadiazon and oxyfluorfen (Table 2). The application of 0.25% or 0.5% surfactant alone did not result in a significant reduction in shoot fresh weight compared with the control. Remarkably, the use of 1% surfactant alone resulted in an approximate 35% reduction in plant biomass, without affecting plant survival. The effective surfactant concentration that increased herbicide activity was different for the two herbicides. When applying oxadiazon with surfactant, shoot biomass declined by to 28 ± 8.0% of untreated controls when applied with 1% surfactant dose and to 9.9 ± 3.6% when applied with 0.5% surfactant (Table 2). When combined with oxyfluorfen, the 0.5% surfactant concentration resulted in a final shoot fresh weight of 6.6 ± 4.4% in comparison to the controls, while the 1% dose brought to a final shoot biomass that was 3.4 ± 3.4% of the untreated control (Table 2).

## 3. Discussion

In this work, plants of two different populations were treated with several herbicides at two growth stages. As evident from the presented data, growth stage had a significant effect on herbicide control of *S. rostratum*. The fact that growth stage has a significant effect on herbicide efficacy is known and was shown for both crop and weed plant species [12,13]. However, as *S. rostratum* may germinate in several germination flashes throughout the season, both young and mature plants can coexist in the same field, thereby reducing the overall efficacy of herbicide treatment. Thus, herbicides that effectively control a wider range of growth stages are more desirable for the farmer.

Surfactants increase herbicide activity as they reduce the droplet surface tension, thus increasing herbicide permeability and mobility through the leaf cuticular layer [14]. Surfactant interactions with both the herbicide and leaf cuticle may differ as a function of their electrical charge (nonionic, ionic and anionic), thereby generating various levels of synergism. Several herbicides, such as the acetolactate inhibitors trifloxysulfuron-methyl [15] and bispyribac-sodium [16], as well as the PPO inhibitor fomesafen [11], require the addition of surfactant to the spraying tank mix. In this study, the effect of surfactant addition on oxadiazon and oxyfluorfen efficacy in larger size (8–9 cm tall) *S. rostratum* plants, was evaluated. Overall, addition of the surfactant induced a synergistic effect, manifesting by higher herbicide activity (Table 2). However, different surfactant concentrations showed diverse synergism levels. For oxadiazon, the lower concentration provided better outcomes, while for oxyfluorfen, the higher surfactant concentration was more effective. Previous studies have demonstrated the synergistic effect of herbicide and surfactant combinations [17,18]. However, evaluation of surfactants as adjuvant should include phytotoxicity tests to ensure crop safety.

One of the most successful integrated weed management approaches is the use of different herbicides in combination with smart crop rotation. Crop rotation is a highly important component of good agricultural practice and can be used to increase productivity and optimize weed control. For instance, nonlegume crop yield can be improved by introducing a legume into the cropping sequence. In comparison to a continuous cotton or wheat-cotton sequence, growing cotton in rotation with vetch (*Vicia sativa* L.), led to a 20% increase in lint yield, even in the absence of N fertilizer [19]. In sugar beet (*Beta vulgaris* L.), yield was 5% higher after introduction of pea (*Pisum sativum* L.), as compared to maize, as a sequence crop [20]. Crop rotation may also be used to reduce weed infestation. A sequence of corn–soybean vs. soybean–soybean crop was shown to decrease *Conyza canadensis* (L.) Cronq. escapes [21]. However, crop rotation with no proper weed management approaches may not be very efficient in reducing weed density [22]. Thus, the chemical management tools that were successful in this study should be combined with adequate crop rotation, in order to reduce *S. rostratum* threat. For instance, using metribuzin in tomato, followed by clopyralid in onion and carfentrazone-ethyl in chickpea, may serve as a long-term crop-herbicide combination that can effectively control *S. rostratum* in these fields. Besides the crops mentioned above, as a summer irrigated crop, corn (*Zea mays* L.) is also an important part of crop rotation in the Israeli agriculture [23]. Although *S. rostratum* shows no marked effect on corn yield, it can be found in high densities at field margins (Figure 1d), where it may enrich the field seed bank for upcoming years. Moreover, this study included herbicides that are registered for use in corn, such as tembutrione. High effectiveness of this herbicide in controlling *S. rostratum*, mainly at the 4–5 cm growth stage, may serve as an advantageous tool to prevent further buildup of *S. rostratum* field populations.

In conclusion, the presented experiments identified several herbicides with varying levels of efficacy, dependent on the plant growth stage, tested on plants from two geographically distinct *S. rostratum* population. In addition, the combination of PPO inhibitors with surfactant increased the susceptibility of *S. rostratum* to herbicides, even at a later growth stage, increasing the overall efficacy of oxadiazon and oxyfluorfen. These findings should be further evaluated under field conditions in order to validate their efficacy. Other approaches, such as nonchemical weed management tools, should also be explored to prevent future damage of agricultural habitats by *S. rostratum*.

## 4. Materials and Methods

### 4.1. Plant Material

As *S. rostratum* seeds possess high dormancy, which may prevent their germination [24], the presented herbicide application tests used field-collected seedlings. Seedling were collected from two fields presenting high infestation of *S. rostratum*, located near Moshav Givat Yoav (GY) (32.8012266548, 35.6977272346) and Kibbutz Ginegar (GO) (32.6543271802, 35.2488696828) (Figure 1). To ensure appropriate representation of the field population, seedlings at the three-four leaf stage were randomly selected from each field population. Seedlings were then transplanted into 250 mL pots filled with commercial potting medium (Tuff, Marom Golan, Israel), including Osmocote^®^ slow-release fertilizer, at the New-Yaar Research Center. Plants were maintained in a greenhouse under Israeli summer conditions and watered daily.

### 4.2. Herbicides Application

Experiments were carried out in the summer of 2020, in a greenhouse at the Newe-Yaar Research Center, under natural conditions. Plants from both GY and GO populations were sprayed when reaching heights of 4–5 cm and 8–9 cm. Experiments were spaced in time such that plants from the first group (4–5 cm) were sprayed two weeks prior to plants from the second group (8–9 cm). Plants were treated with eight different herbicides at the recommended field rate as specified in Table 3, using a chain-driven sprayer delivering 300 L ha^−1^, with a flat-fan 8001E nozzle (TeeJet^®^, Spraying Systems Co., Wheaton, IL, USA). All experiments were arranged in a fully randomized-factorial design, with eight to ten replicates for each treatment. Shoot fresh weight of each plant was recorded 21 days after treatment (DAT).

### 4.3. Surfactant Effect

Both oxyfluorfen and oxadiazon, were applied in a tank mix with the nonionic surfactant alkyl phenol ethylene oxide (Shatah 90^®^, ADAMA-Makhteshim, Israel). The effect of increasing surfactant concentrations on *S. rostratum* control was evaluated. Plants from both populations, GY and GO, reacted similarly to oxadiazon at heights of 8–9 cm (Table 1). However, plants from the GY population were less responsive to oxyfluorfen at the later growth stage. Thus, advanced growth stage plants from the GY population were chosen for this experiment. Plants were treated with three different surfactant concentrations (0.25%, 0.5% and 1%), applied with or without oxyfluorfen and oxadiazon (at recommended field rate; Table 3). Experiments were conducted as specified above. Shoot fresh weight and survival rate were recorded for each plant 21 DAT.

### 4.4. Statistical Analyses

Shoot fresh weight data were analyzed using ANOVA in JMP (ver. 15) statistical package (SAS Institute Inc., Cary, NC, USA). Data was visualized using SigmaPlot (ver. 13) software (Systat Software Inc., San Jose, CA, USA). The assumptions of homoscedasticity and normality were met using Levene′s tests. For both experiments, i.e., the response of GO and GY populations to herbicide treatments at different growth stages and the synergistic effect of surfactant application, interactions between experimental parameters were observed. Thus, data from each experiment were analyzed separately as experiment-by-treatment. For all experiments, shoot fresh weight was analyzed as percent of untreated control. All parameters were subjected to one-way ANOVA and means were separated using the Tukey-HSD test (*α* = 0.05).

## Figures and Tables

**Table 1:** Differences in mean shoot fresh weight among *Solanum rostratum* populations in response to various herbicides.

		Mean Shoot Fresh Weight (% of Control)		
Population ^a^	Herbicide ^b^	4–5 cm ^c^	8–9 cm	*p*-Value
GY	Carfentrazone-ethyl	0.57	0.32	0.2567
	Clomazone	36.34	87.56	<0.0001
	Clopyralid	60.09	98.00	<0.0001
	Metribuzin	0.89	47.75	<0.0001
	Oxadiazon	3.01	30.44	<0.0001
	Oxyfluorfen	4.77	48.75	<0.0001
	Rimsulfuron	87.84	56.00	0.0008
	Tembotrione	4.61	47.56	<0.0001
GO	Carfentrazone-ethyl	0.22	0.19	0.8502
	Clomazone	79.52	103.34	0.0084
	Clopyralid	42.54	88.89	<0.0001
	Metribuzin	6.42	75.93	<0.0001
	Oxadiazon	5.65	39.69	<0.0001
	Oxyfluorfen	2.76	33.13	<0.0001
	Rimsulfuron	93.83	110.08	0.0134
	Tembotrione	10.76	75.31	<0.0001

^a^ GY—Givat Yoav and GO—Ginegar; ^b^ All herbicides were applied at the labeled field rate as specified in Table 3; ^c^ Plants were sprayed at two different growth stages (4–5 cm and 8–9 cm); Shoot fresh weight was recorded 21 days after herbicide application.

**Table 2:** Reduction of shoot fresh weight (mean ± SE) and survival rate of 8–9 cm tall *Solanum rostratum* Givat Yoav plants following oxadiazon and oxyfluorfen application, with or without surfactant.

Treatment ^a^	Mean (SE) ^b^	Lower 95%	Upper 95%	Survival (%)
0.5% surfactant	103.40 (7.30) a	84.65	122.16	100%
Control	102.14 (5.51) a	89.67	114.61	100%
0.25% surfactant	94.04 (9.04) ab	71.92	116.16	100%
1% surfactant	65.50 (4.89) b	53.52	77.48	100%
Oxadiazon + 1% surfactant	28.00 (8.01) c	9.87	46.13	70%
Oxadiazon + 0.25% surfactant	26.18 (6.41) c	11.69	40.68	80%
Oxifluorfen	25.54 (9.73) c	2.54	48.54	50%
Oxadiazon	19.38 (7.26) c	2.94	35.81	50%
Oxadiazon + 0.5% surfactant	9.84 (3.54) c	1.84	17.85	40%
Oxifluorfen + 0.5% surfactant	6.57 (4.42) c	−3.63	16.77	30%
Oxifluorfen + 0.25% surfactant	4.10 (4.08) c	−5.14	13.34	20%
Oxifluorfen + 1% surfactant	3.38 (3.36) c	−4.37	11.13	20%

^a^ All herbicides were applied at the labeled field rate as specified in Table 3; ^b^ Different lowercase letters indicate statistically significant differences among treatments, as determined by a Tukey–Kramer HSD test (α = 0.05); Shoot fresh weight was recorded 21 days after herbicide application.

**Table 3:** List of herbicides used in this study and their labeled field rates.

Common Name	Trade Name	MOA ^a^	Manufacturer	Rate (g ai ha^−1^)
Oxyfluorfen	Galigan^®^	PPO	ADAMA-Agan	352.5
Carfentrazone-ethyl	Spotlight^®^	PPO	FMC	0.9
Oxadiazon	Ronstar^®^	PPO	Bayer	875
Clopyralid	Lontrel^®^	Auxinic herbicide	Corteva	50
Clomazone	Comand^®^	Carotenoid biosynthesis	FMC	540
Tembotrione	Laudis^®^	HPPD	Bayer	99
Metribuzin	Sencor^®^	PSII	Bayer	175
Rimsulfuron	Titus^®^	ALS	Corteva	25 ^b^

^a^ Mode of action; ^b^ Addition of a nonionic surfactant (Shatah 90^®^, ADAMA-Makhteshim, 0.05%) as part of the manufacturer recommendation.
